# An Onset Detection Method for Slowly Activated Muscle Based on Marginal Spectrum Entropy

**DOI:** 10.3390/s25102963

**Published:** 2025-05-08

**Authors:** Xiaolei Huang, Jinzhuang Xiao, Qing Chang, Bin Fang

**Affiliations:** 1 College of Electrical Engineering, Hebei University of Architecture, Zhangjiakou 075000, China; hxl2103@hebiace.edu.cn (X.H.); cq1066@hebiace.edu.cn (Q.C.); fb2077@hebiace.edu.cn (B.F.); 2 College of Eletronic and Information Engineering, Hebei University, Baoding 071000, China

**Keywords:** muscle activation, slow activation, Hilbert–Huang transform (HHT), marginal spectrum entropy (MSE)

## Abstract

Muscle activity is composed of fast and slow activations. The detection of the onset time of the electromyogram signal, which is slowly activated, is difficult. This paper proposes a detection method based on marginal spectral entropy (MSE). The surface electromyography (sEMG) signal of the soleus during normal walking was collected by a wireless electromyography acquisition system. The proposed MSE-based detection method is based on the Hilbert–Huang transform (HHT) combined with information entropy. By comparing the changes in MSE before and after muscle activation to plot a trend line, the point of fastest change on the trend line was defined as the onset time of muscle activation. This method was compared with the amplitude threshold method and the Teager–Kaiser energy (TKE) operator method. The results show that the onset time of muscle activation detected by this method is 0.14 s earlier than the amplitude threshold method and 0.16 s earlier than the TKE operator method. The detection results were significantly different (*p* < 0.05), indicating that this method has higher detection accuracy for the onset time of the sEMG signal, which is slowly activated.

## 1. Introduction

Electromyographic (EMG) signals reflect the electrical activity generated by muscle contractions and are widely used in motor rehabilitation [1], bionics [2], and sports science [3]. Surface electromyography (sEMG), obtained non-invasively via skin surface electrodes, provides essential information about muscle function, including the timing, intensity, and regularity of muscle activation [4]. In clinical settings, the precise determination of muscle activation onset is critical for diagnosing neuromuscular dysfunctions and guiding rehabilitation strategies in conditions such as stroke [5], cerebral palsy [6], and Parkinson’s disease [7]. It can also aid in the assessment of aging-related co-activation patterns during locomotion [8] and support the design of neural prostheses [9].

A fundamental challenge in sEMG signal analysis is the accurate detection of the onset time of muscle activation. Traditional methods, such as the amplitude threshold approach [10] and the Teager–Kaiser energy (TKE) operator [11], typically rely on preset empirical thresholds or energy criteria. Although these methods are simple and computationally efficient [12], they are highly dependent on user-defined parameters. In particular, they often fail to detect slow activations, where the muscle’s electrical activity increases gradually. This situation is common in muscles with a predominantly slow-twitch fiber composition, such as the soleus, whose activation is subtle yet essential for maintaining posture and balance [13]. The role of the soleus in postural control has been emphasized in biomechanical studies [14], and its physiological characteristics have been extensively reviewed [15].

Given the challenges of detecting slow muscle activations, several recent studies have explored entropy-based approaches and nonlinear signal analysis techniques to better capture the complex dynamics of sEMG signals. For example, marginal spectrum entropy (MSE) has been proposed as an effective time–frequency representation that integrates the Hilbert–Huang transform (HHT) with information entropy [16]. Related work has demonstrated the feasibility of using MSE to characterize neuromuscular patterns in clinical applications [17]. However, many entropy-based methods from 2021 to 2024 either focus primarily on fast activation phenomena or rely on supervised deep learning frameworks [18], which require large labeled datasets and significant computational resources [19], making them difficult to generalize in real-world settings.

In light of these limitations, this paper introduces a novel detection method based on marginal spectrum entropy. By combining HHT with entropy analysis, the proposed method captures subtle changes that occur during slow muscle activation without relying on fixed amplitude thresholds. It is specifically designed to reduce false detections and missed onsets associated with conventional techniques [20].

This study aims to address the key challenge of accurately detecting the onset of slowly activated surface electromyographic (sEMG) signals, which are commonly observed in muscles dominated by slow-twitch fibers (e.g., the soleus). These signals typically exhibit low-amplitude, gradually increasing activation patterns that are difficult to detect using conventional methods. The main objectives of this study are summarized as follows:

1. **Improve the accuracy of onset detection in slowly activated sEMG signals:** Slowly activated signals, typically observed in slow-twitch muscles such as the soleus, exhibit smooth amplitude transitions with shallow rising slopes. Such characteristics often lead to delayed or missed detections when using traditional methods based on amplitude thresholds or energy criteria. By identifying the point of steepest decline in the marginal spectrum entropy (MSE) trend, the proposed method captures the most prominent spectral transition and provides a physiologically meaningful estimate of activation onset.
2. **Reduce reliance on subjective thresholding:** Traditional methods often depend on manually set thresholds, which are sensitive to individual variability, background noise, and baseline drift, resulting in poor robustness and reproducibility. The proposed method, based on entropy modeling, is adaptive and unsupervised, thereby eliminating the need for manual tuning and improving generalizability across subjects and conditions.
3. **Ensure applicability across a wide range of neuromuscular conditions:** The method is applicable not only to healthy subjects but also to clinical populations with neuromuscular impairments such as stroke, Parkinson’s disease, or muscular atrophy. In addition to onset detection, it can support real-time muscle monitoring, motor intention decoding, brain–computer interface (BCI) control, and rehabilitation evaluation.
4. **Support real-time intention recognition and rehabilitation interventions:** Experimental results show that the proposed method detects activation, on average, 0.14 to 0.16 s earlier than conventional approaches. Although numerically small, this lead time is critical in time-sensitive applications such as gait control and wearable device feedback, where milliseconds matter. Early detection enables more responsive system behavior, enhances motion naturalness, and reduces the cognitive and physical burden on the user.
5. **Enhance the interpretability of the “slow activation” process:** “Slow activation” refers not only to gradual amplitude changes but also to smooth transitions in both temporal and spectral domains, often lacking distinct boundaries. Such signals are more susceptible to noise and individual variability, and are frequently misclassified by traditional algorithms. The proposed MSE-plus-slope fusion mechanism is sensitive to weak yet continuous trends, enabling the identification of nuanced neuromuscular modulation under fatigue, co-contraction, or pathological conditions.

## 2. Related Work

Surface electromyography (sEMG) is a widely used noninvasive technique for assessing neuromuscular activity, and accurate detection of its onset timing is critical in various domains such as motor rehabilitation, neurological disorder diagnosis, and human–machine interaction. However, detecting the onset of slowly activated muscle signals remains particularly challenging due to their smooth rise profiles and low amplitudes. Conventional energy- or threshold-based methods often suffer from false or missed detections under these conditions. To address these challenges, researchers have proposed a variety of improvements aimed at enhancing detection performance under low signal-to-noise ratio (SNR) and non-stationary conditions. These studies can be broadly categorized into conventional methods, entropy-based approaches, and extensions based on marginal spectrum entropy (MSE).

### 2.1. Conventional sEMG Onset Detection Techniques

Early sEMG onset detection methods primarily relied on threshold-based strategies. Carvalho et al. (2023) conducted a systematic evaluation of three common approaches, that is, single threshold (ST), double threshold (DT), and adaptive threshold (AT). While ST remains widely adopted due to its simplicity, it is prone to noise-induced errors. DT introduces a temporal constraint to enhance robustness, and AT leverages dynamic adjustment via sliding windows to improve accuracy [21]. Despite their relative strengths, these methods remain inadequate for detecting slowly activated signals with ambiguous boundaries. To mitigate the influence of electrocardiographic (ECG) artifacts, Zhou and Zhang (2013) introduced a detection strategy based on sample entropy (SampEn), which identifies signal onset by capturing changes in signal complexity rather than amplitude alone [22]. This approach significantly improves robustness in low-SNR scenarios. Further addressing SNR sensitivity, Solnik et al. (2008, 2010) incorporated the Teager–Kaiser energy operator (TKEO) to enhance signal features, achieving a substantial reduction in detection error and heightened responsiveness to abrupt changes [23,24]. TKEO consistently improved performance across various classifiers, including GLRT and manual judgment. In a complementary line of work, Benazzouz and Hadj (2018) applied discrete wavelet transform (DWT) with soft/hard thresholding to extract multiscale features and suppress baseline drift and spurious activations [25]. Their approach achieved near-manual accuracy in a cohort of 20 subjects, demonstrating good real-time applicability in dynamic motion settings. While traditional techniques offer computational efficiency and real-time feasibility, their reliance on fixed heuristics renders them less effective in handling non-stationary, low-amplitude, and slowly evolving signals. This limitation motivates the exploration of more robust nonlinear modeling strategies.

### 2.2. Entropy-Based Approaches in sEMG Analysis

To better characterize the nonlinear and non-stationary nature of sEMG signals, recent studies have explored entropy-based features for onset detection. Hu et al. (2018) proposed a hybrid framework incorporating Hilbert–Huang transform (HHT) and marginal spectrum entropy (MSE), which uses adaptive thresholding on dynamic entropy curves to detect muscle activation with high precision [26]. Their method achieved robust performance in clinical assessments of stroke patients. Targeting spurious spike artifacts in voluntary muscle activity, Zhang and Zhou (2012) introduced a SampEn-based detection method that outperforms both amplitude thresholding and TKEO, particularly in noise-contaminated conditions [27]. To further reduce parameter sensitivity and enhance applicability in short time windows, Lyu et al. (2014) developed a fuzzy entropy (FuzzyEn) approach that demonstrated fast and accurate detection across multiple rehabilitation modalities [28]. Compared to conventional methods, entropy-based strategies are data-driven and complexity-aware, allowing for more reliable detection of subtle changes in neuromuscular activity without reliance on predefined amplitude models. This makes them particularly advantageous for capturing slow, weak signal transitions.

### 2.3. Marginal Spectrum Entropy: Principles and Limitations

As a key variant within the entropy family, marginal spectrum entropy (MSE) integrates HHT decomposition with Shannon entropy to describe spectral energy redistribution over time. Hu et al. (2018) utilized MSE combined with root mean square (RMS) deviation as a dual-index detection strategy to identify stretch reflex onsets (SROs) in stroke patients [26]. Their method exhibited excellent test-retest reliability (ICC = 0.914) and predictive consistency with the MAS score, confirming its clinical utility. To improve detection under high-noise conditions, Du et al. (2019) proposed a hybrid model based on empirical mode decomposition (EMD) and modified SampEn. By selecting intrinsic mode functions (IMFs) with high correlation to the original signal and suppressing spurious activation through soft thresholding, the method achieved a 28% increase in detection accuracy on both simulated and real signals, outperforming standalone SampEn and wavelet-based approaches [29]. Despite the advantages of MSE in capturing dynamic spectral patterns, its implementation often involves heavy dependence on window parameters and high computational costs associated with EMD. As the demand for real-time and edge-deployable solutions increases, future work must focus on simplifying the architecture and optimizing inference efficiency. Overall, a paradigm shift is underway—from rule-based to complexity-driven detection frameworks. The method proposed in this study, which incorporates trend slope analysis of MSE, is positioned within this evolution and aims to address the precision and generalization limitations of conventional approaches for slow-onset detection.

## 3. Materials and Method

### 3.1. Data Collection

A total of 20 soleus sEMG signal sets were collected from 10 male and 10 female healthy participants during walking. All signals were acquired and preprocessed under standardized conditions to ensure temporal integrity and signal quality. Detailed data for the 20 sets are presented in Table 1. Detailed data for the male participants are in Table 2, and data for the female participants are in Table 3. A total of 20 healthy participants were recruited, and high-resolution sEMG signals from the soleus muscle during natural walking were collected at a sampling rate of 1500 Hz. Although limited in size, this dataset is representative of early-phase algorithm validation and serves as a foundation for future generalization.

### 3.2. Data Annotation and Preprocessing Procedures

In this study, a systematic data preprocessing pipeline was applied to ensure the validity and comparability of the acquired sEMG signals. First, a band-pass filter ranging from 20 to 450 Hz was used to remove power line interference and low-frequency drift while preserving the main frequency components of the muscle activity. Then, a fifth-order zero-phase Butterworth filter was applied to further smooth the signal without introducing phase distortion. After filtering, the signals were segmented into overlapping sliding windows of 128 ms in length with a 50% overlap rate. Each window served as an independent analysis unit for the calculation of marginal spectrum entropy (MSE) and trend slope features. To enhance consistency across samples, Z-score normalization was applied to all windows, standardizing the data to zero mean and unit variance, thus minimizing the influence of amplitude variations during feature extraction. In addition, to validate the effectiveness of the proposed method, reference onset labels were generated through a combination of manual annotation and expert verification. Initial annotations were made based on the visual inspection of sEMG waveform patterns and abrupt changes in muscle activity. These annotations were then reviewed and cross-validated by two experts with backgrounds in biomechanics. The resulting consensus labels served as the ground truth for supervised evaluation, providing a reliable benchmark for assessing detection accuracy, detection advance time, RMSE, and other performance metrics.

### 3.3. Proposed Method

**Figure 1:**
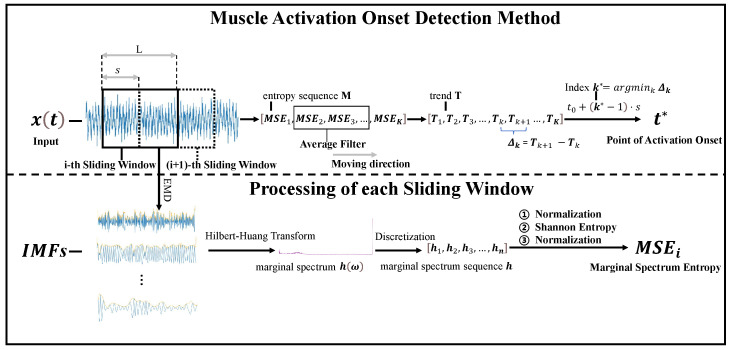
Overview of the muscle activation onset detection method based on marginal spectrum entropy (MSE) and Hilbert–Huang transform (HHT). The upper part illustrates the overall pipeline, where the raw sEMG signal $x\left(t\right)$ is segmented into overlapping windows with a length *L* and step size *s*. Each window is processed to compute the MSE, forming an entropy sequence M, which is smoothed to obtain a trend signal T. The onset time $t^{*}$ is estimated by locating the point with the steepest entropy drop. The lower part details the processing within each window: the empirical mode decomposition (EMD) yields intrinsic mode functions (IMFs), which are transformed by the Hilbert–Huang method to obtain the marginal spectrum h(ω). Following discretization, the marginal spectrum sequence h is derived. Subsequently, the sequence is first normalized to obtain a probability distribution, from which the Shannon entropy is calculated. Finally, the entropy value is normalized to produce the marginal spectrum entropy (MSE) for the current window.

This section introduces a muscle activation onset detection method specifically designed for slowly activated surface EMG (sEMG) signals. The proposed approach integrates the Hilbert–Huang transform (HHT) for adaptive time–frequency decomposition and employs marginal spectrum entropy (MSE) to characterize the temporal evolution of spectral complexity. Within a sliding window framework, the method analyzes the trend and slope of the MSE sequence to robustly identify the activation onset without relying on predefined amplitude thresholds. This strategy is particularly effective in detecting subtle neuromuscular transitions that are often missed by traditional energy- or threshold-based techniques. The overall framework comprises three key stages, as follows: (1) the Hilbert–Huang transform; (2) HHT-based marginal spectrum entropy; and (3) muscle activation onset detection based on marginal spectrum entropy. The complete pipeline of the proposed method is illustrated in Figure 1 and Algorithm 1.
**Algorithm 1:** Muscle activation onset detection based on MSE and HHT
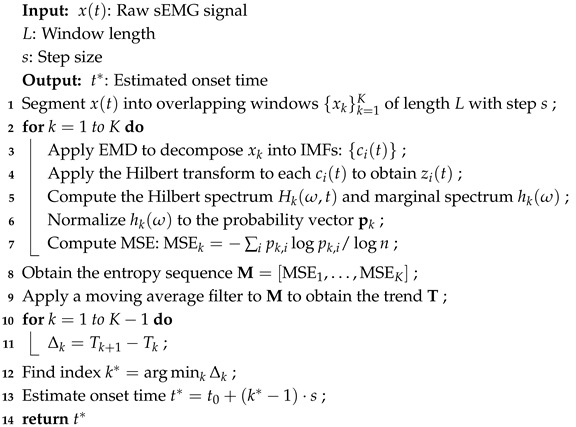


#### 3.3.1. Hilbert–Huang Transform

The Hilbert–Huang transform (HHT), proposed by Huang et al. [30], is an adaptive signal analysis method specifically designed for nonlinear and non-stationary time series. It consists of two sequential components: empirical mode decomposition (EMD) and Hilbert transform. In this study, HHT is applied to decompose surface electromyographic (sEMG) signals into intrinsic oscillatory components, from which time–frequency information is extracted for subsequent entropy analysis. Given a raw sEMG signal x(t), EMD is first performed to decompose it into a sum of *M* intrinsic mode functions (IMFs) $c_{i}\left(t\right)$ and a final residual component $r_{M}\left(t\right)$ such that

$$x\left(t\right)=\sum_{i=1}^{M}c_{i}\left(t\right)+r_{M}\left(t\right).$$

Each IMF $c_{i}\left(t\right)$ must satisfy the following two constraints: (1) the number of extrema and zero-crossings must either be equal or differ by no more than one, and (2) at any time point, the local mean value—defined by the average of the upper and lower envelopes interpolated from local maxima u(t) and minima l(t)—must be zero. The local mean m(t) is computed as follows:

$$m\left(t\right)=\frac{u(t)+l(t)}{2},$$

and is subtracted from the signal to obtain the detail component

h(t)=x(t)−m(t).

If h(t) meets the definition of an IMF, it is designated as $c_{1}\left(t\right)$; otherwise, the sifting process is iteratively repeated until convergence. The first residual is then computed by removing $c_{1}\left(t\right)$ from the original signal:

$$r_{1}\left(t\right)=x\left(t\right)-c_{1}\left(t\right),$$

The same process is applied to $r_{1}\left(t\right)$ to extract the subsequent IMFs. This recursive decomposition continues until the residual becomes a monotonic function or a signal with only one extremum. Once all IMFs are obtained, the Hilbert transform is applied to each IMF $c_{i}\left(t\right)$ to generate its analytic signal:

$$z_{i}\left(t\right)=c_{i}\left(t\right)+j\cdot H\left\{c_{i}\left(t\right)\right\},$$

where H{·} denotes the Hilbert transform operator. From this, the instantaneous amplitude $A_{i}\left(t\right)$ and instantaneous phase $\phi_{i}\left(t\right)$ can be derived:

$$A_{i}\left(t\right)=\left|z_{i}\left(t\right)\right|,\;\phi_{i}\left(t\right)=\arg\left(z_{i}\left(t\right)\right),$$

leading to the computation of the instantaneous frequency as

$$\omega_{i}\left(t\right)=\frac{d\phi_{i}\left(t\right)}{dt}.$$

The time–frequency–energy distribution of each IMF can then be represented as a Hilbert spectrum H(ω,t), which describes the amplitude (or energy) contribution at each time *t* and frequency ω. To provide a compact frequency-domain representation over the entire time period [0,T], the marginal spectrum is constructed by integrating the Hilbert spectrum along the time axis:

$$h\left(\omega \right)=\int_{0}^{T}H\left(\omega ,t\right)\;dt.$$

The resulting marginal spectrum h(ω) reflects the cumulative amplitude contribution of each frequency across the entire signal duration. This spectrum preserves the intrinsic time–frequency structure of the signal and serves as the basis for the marginal spectrum entropy calculation described in Section 2.2.

#### 3.3.2. HHT-Based Marginal Spectrum Entropy

Marginal spectrum entropy (MSE) is employed in this study to characterize the complexity and dynamic variation of sEMG signals in the frequency domain after the Hilbert–Huang decomposition. Unlike time-domain energy measures or threshold-dependent metrics, MSE provides a normalized, statistical description of how signal energy is distributed across frequencies and is highly sensitive to subtle changes induced by neuromuscular activation.

Given a discretized marginal spectrum $h=[h_{1},h_{2},\ldots ,h_{n}]$, where $h_{i}$ denotes the amplitude corresponding to the *i*-th frequency bin, a probability distribution over the frequency axis is constructed by normalizing each component:

$$p_{i}=\frac{h_{i}}{\sum_{k=1}^{n}h_{k}},\;\mathrm{for}\;i=1,2,\ldots ,n.$$

Here, $p_{i}$ represents the relative spectral energy at the *i*-th frequency, satisfying the property $\sum_{i=1}^{n}p_{i}=1$. Using this probability vector $p=[p_{1},p_{2},\ldots ,p_{n}]$, the spectral entropy is defined based on Shannon entropy as follows:

$${\mathrm{MSE}}_{\mathrm{raw}}=-\sum_{i=1}^{n}p_{i}\log p_{i}.$$

In order to constrain the entropy value to a standardized range and account for different frequency resolutions, the raw entropy is normalized by its maximum possible value logn, where *n* is the number of frequency bins:

$$\mathrm{MSE}=\frac{{\mathrm{MSE}}_{\mathrm{raw}}}{\log n},\;\mathrm{with}\;\mathrm{MSE}\in \left[0,1\right].$$

A low MSE value indicates that the frequency energy is concentrated in a small number of bins (i.e., the signal is spectrally regular), whereas a high MSE value implies that the energy is dispersed across many frequency components, reflecting increased complexity or dynamical transitions in the signal. In the context of muscle activation detection, the evolution of MSE over time windows is monitored. When a muscle transitions from a resting to an active state, it induces a redistribution of frequency energy, which manifests as a rapid rise or drop in MSE. This property is leveraged in the next section, where the point of maximum slope change in the MSE sequence is identified as the activation onset.

#### 3.3.3. Muscle Activation Onset Detection Based on Marginal Spectrum Entropy

To detect the onset of muscle activation from the processed sEMG signal, a sliding-window approach is applied to compute the temporal evolution of marginal spectrum entropy (MSE). The main assumption is that muscle activation induces spectral redistribution, which manifests as a sudden change in the MSE values over time. Let the filtered sEMG signal be x(t), and let it be segmented into overlapping windows of fixed length *L* and step size *s*. For each window indexed by *k*, the corresponding segment $x_{k}$ is used to compute the marginal spectrum entropy ${\mathrm{MSE}}_{k}$ as described in Section 2.2. This yields a sequence of entropy values:

$$M=\left[{\mathrm{MSE}}_{1},{\mathrm{MSE}}_{2},\ldots ,{\mathrm{MSE}}_{K}\right],$$

where *K* is the total number of windows. To smooth out local fluctuations and reduce noise sensitivity, a moving average filter is applied to M, resulting in a trend sequence $T=[T_{1},T_{2},\ldots ,T_{K}]$. To determine the activation point, we analyze the slope of this trend sequence. The first-order difference of the trend is computed as follows:

$$\Delta_{k}=T_{k+1}-T_{k},\;\mathrm{for}\;k=1,2,\ldots ,K-1.$$

The onset of muscle activation is defined as the point $k^{*}$ where the slope reaches its most negative value:

$$k^{*}=\arg\min_{k}\Delta_{k}.$$

**Figure 2:**
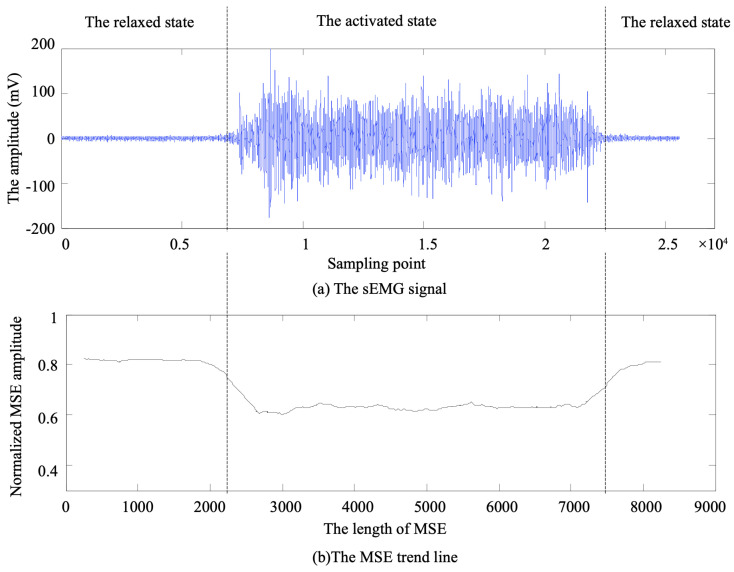
The sEMG signal and the MSE trend line: (**a**) the sEMG signal; (**b**) the MSE trend line.

Finally, the estimated onset time $t^{*}$ is computed by the following:

$$t^{*}=t_{0}+\left(k^{*}-1\right)\cdot s,$$

where $t_{0}$ is the starting time of the signal. This method eliminates the need for manually defined amplitude thresholds and provides a data-driven alternative based on spectral complexity. It is particularly effective for detecting slowly activated muscle contractions that may not produce distinct amplitude spikes. Figure 2 illustrates the process of onset detection using the MSE trend curve and the estimated activation point. It should be noted that the proposed method operates in a post-processing mode after the entire sEMG sequence is collected. Real-time deployment would require the redesign of the entropy estimation and trend slope computation to function incrementally.

### 3.4. Experimental Setup

**Figure 3:**
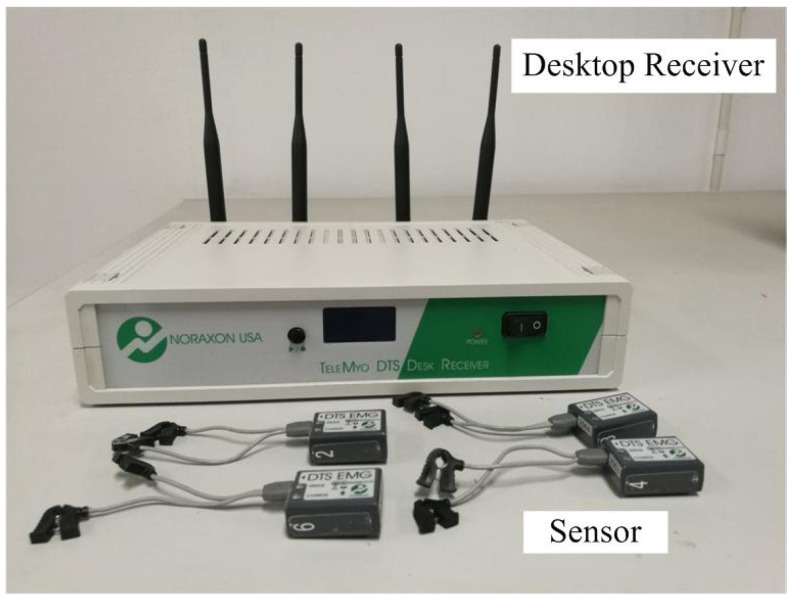
Wireless sEMG acquisition device used in the experiment.

**Figure 4:**
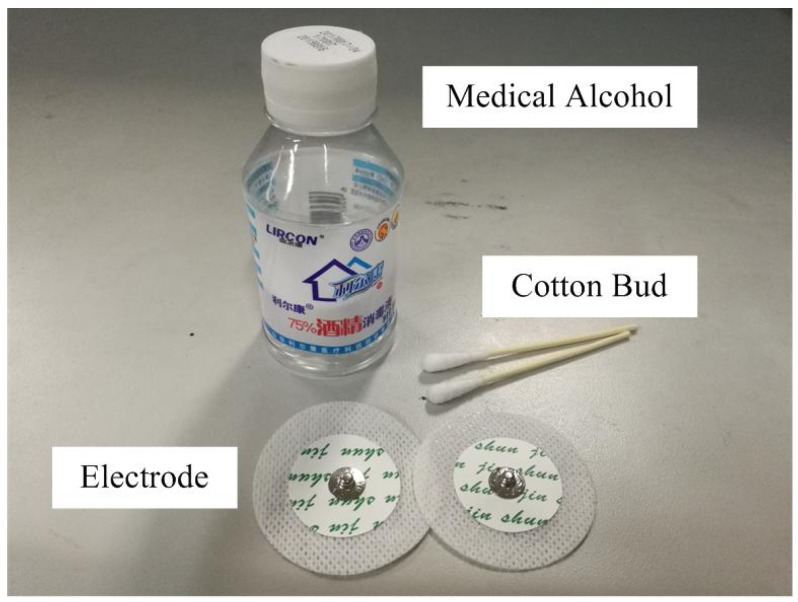
Consumables including disposable electrodes.

To verify the effectiveness of the proposed muscle activation detection method based on marginal spectrum entropy (MSE), we conducted experiments using high-resolution surface EMG (sEMG) data collected from healthy subjects during walking. All signals were acquired using the NORAXON wireless sEMG acquisition system, which samples at a frequency of 1500 Hz. Prior to the experiment, the acquisition devices were calibrated, and participants were instructed to follow standardized movement procedures to ensure data quality and consistency, as shown in Figure 3 and Figure 4.

A total of 20 healthy college students (10 male and 10 female, all right-foot dominant) participated in the study. None had experienced muscle injuries or engaged in high-intensity exercise within 48 h prior to recording. Participants’ anthropometric details, including average age, height, and weight, are summarized in Table 4.

**Figure 5:**
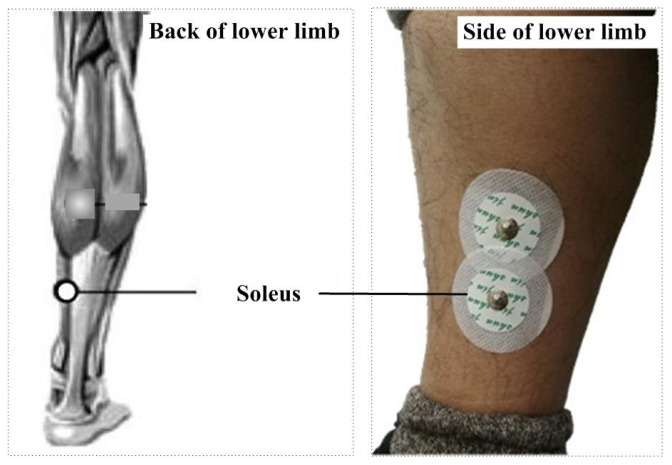
The positions of the soleus and electrodes.

Electrodes were placed on the soleus muscle of the right leg, a muscle primarily composed of slow-twitch fibers, as shown in Figure 5. Skin preparation was performed using abrasive paste and alcohol to reduce impedance. Electrode positions followed SENIAM recommendations and were aligned with the muscle fiber orientation to ensure high signal fidelity. Electrodes were fixed using medical-grade adhesive and elastic bandages. Participants walked naturally along a straight path while sEMG signals were continuously recorded. Each subject performed several walking trials, and a total of 20 representative sEMG sequences were extracted for analysis. These recordings were specifically selected to capture the transition of the soleus muscle from relaxation to slow activation during the gait cycle, providing ideal conditions for validating the MSE-based detection framework. This study focuses on slow-activation muscle onset detection; thus, the soleus muscle, predominantly composed of slow-twitch fibers, was selected. In the software context, this choice enables testing under low-gradient signal conditions, ideal for evaluating the sensitivity of the proposed method to gradual spectral changes.

## 4. Results and Discussion

### 4.1. Performance Evaluation of Five Methods for sEMG Onset Detection

To evaluate the effectiveness of the proposed muscle activation onset detection method based on marginal spectrum entropy (MSE), a series of experiments was conducted using surface electromyography (sEMG) signals collected from the soleus muscle during natural walking. A total of 20 valid sEMG sequences were analyzed and compared against four widely used baseline methods, as follows:

1. Proposed method: Based on marginal spectrum entropy and trend slope analysis.
2. Threshold: Fixed threshold method, where activation is marked when the signal exceeds 30% of its maximum amplitude [31].
3. TKE: Detection based on the Teager–Kaiser energy operator [32].
4. RMS: Root-mean-square (RMS) windowing strategy [33].
5. Wavelet: Wavelet-based onset detection via energy redistribution, as described in [34].

**Figure 6:**
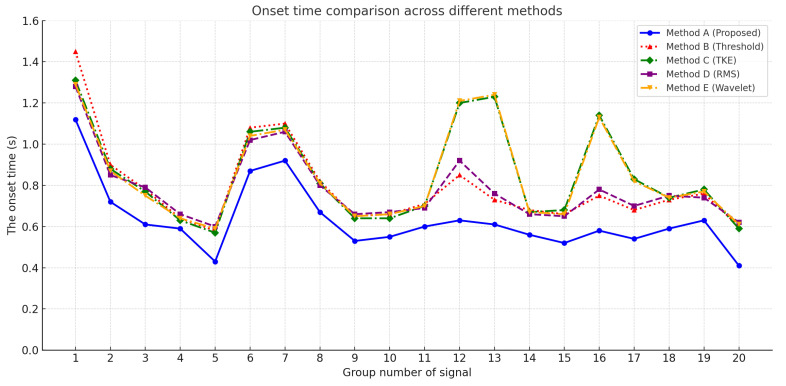
Comparison of onset detection times across 20 sEMG samples using five methods: proposed method (proposed), Method B (threshold-based), Method C (TKE), Method D (RMS), and Method E (wavelet). The proposed method demonstrates superior early detection performance.

As shown in Figure 6, the onset times detected by all five methods are plotted across 20 signal groups. It is evident that the proposed method consistently achieves earlier activation detection in most cases compared to the alternatives.

**Figure 7:**
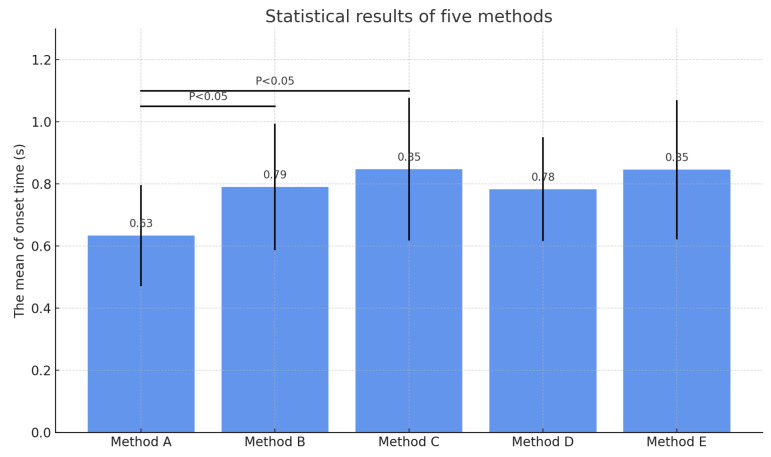
Mean and standard deviation of onset detection times for five methods. Method A (proposed method) demonstrates statistically earlier detection compared to other methods (p<0.05).

To further compare the methods in terms of average performance, a statistical bar chart was generated, as shown in Figure 7. Each bar represents the mean onset time for the corresponding method, with error bars indicating standard deviations. Statistical significance annotations (p<0.05) were added to highlight differences between the proposed method and other methods.

As shown in Figure 7, the proposed method not only achieves the earliest detection on average but also exhibits the smallest variance, indicating higher stability and consistency. In order to quantitatively assess detection performance, multiple evaluation metrics were computed, including sensitivity, specificity, false negative rate (FNR), false positive rate (FPR), root mean square error (RMSE), and detection advance. The results are summarized in Table 5.

The proposed method outperforms all baselines across the listed metrics, especially in FNR and FPR, indicating a significantly lower probability of both missed detections and false positives. Moreover, the lowest RMSE suggests enhanced temporal precision in estimating onset time. From a clinical perspective, although an advance of 0.14–0.16 s may appear small in absolute terms, it can be crucial for real-time rehabilitation systems. For instance, in post-stroke gait training or wearable exoskeleton control, early identification of muscle activation enables faster system response, more synchronized actuation, and reduction of fatigue or coordination delays. The proposed approach thus holds practical value for both engineering applications and clinical interventions. While Method B and Method C can, in theory, perform real-time detection based on instantaneous features, the proposed MSE method is currently designed for offline analysis with a full signal window.

### 4.2. Ablation Study Analysis

To further evaluate the effectiveness of each core component within the proposed model, two ablation experiments were conducted. These experiments were designed to assess the contributions of the marginal spectrum entropy (MSE) module and the slope-based trend analysis module to the overall detection performance. In the first ablation setting, the MSE computation was removed and replaced by a simple moving average of signal energy within the same sliding window. The activation onset was then identified using the slope analysis of the energy signal. In the second ablation setting, the slope-based detection component was eliminated, and the onset was directly determined by identifying the minimum point on the MSE curve, without calculating the temporal slope. The simplified variants were compared against the complete model to evaluate the independent contributions of each module.

As shown in Table 6, the full model achieved the best performance across all metrics, including the highest sensitivity (0.92), specificity (0.89), and the lowest false negative rate (FNR = 0.08) and false positive rate (FPR = 0.11). The full configuration also attained the lowest root mean square error (RMSE = 0.041 s) and the most advanced average detection lead time (0.14 s).

**Figure 8:**
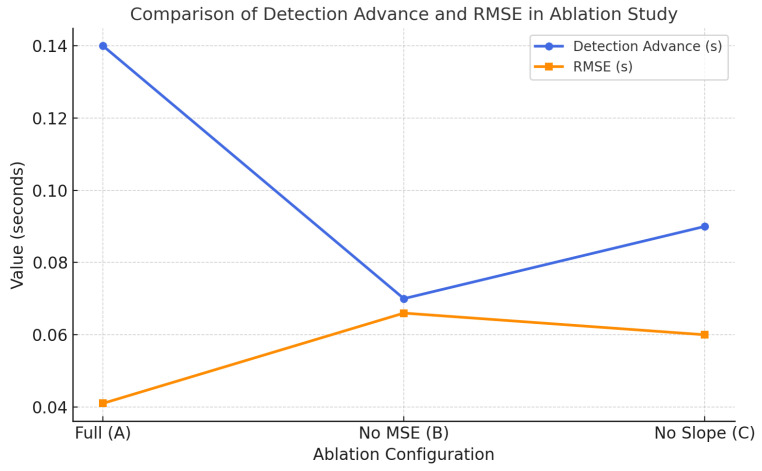
Comparison of detection advance and RMSE under three ablation configurations. The full model achieves the best trade-off in both timeliness and accuracy.

To provide a more intuitive comparison of the effects of different ablation configurations, Figure 8 illustrates the variations in detection advance and RMSE across the three configurations. As observed, the full model (denoted as Full) consistently outperformed the two simplified variants in both metrics, with a detection advance of 0.14 s and a minimal RMSE of 0.041 s.

Based on the combined analysis of Table 6 and Figure 8, both the MSE and slope modules are found to be critical to the model’s effectiveness. Removing the MSE component results in a substantial drop in sensitivity and an increase in detection error, indicating that spectral entropy plays a vital role in capturing the frequency complexity changes associated with slow muscle activations. Similarly, eliminating the slope-based detection module leads to a degradation in temporal localization accuracy, with RMSE increasing from 0.041 to 0.060 s, suggesting that the trend analysis contributes significantly to precise onset estimation. The integration of both modules substantially enhances the model’s responsiveness and robustness, particularly in handling gradually activating muscle signals.

#### 4.2.1. Validation of the Sliding Window Mechanism

**Figure 9:**
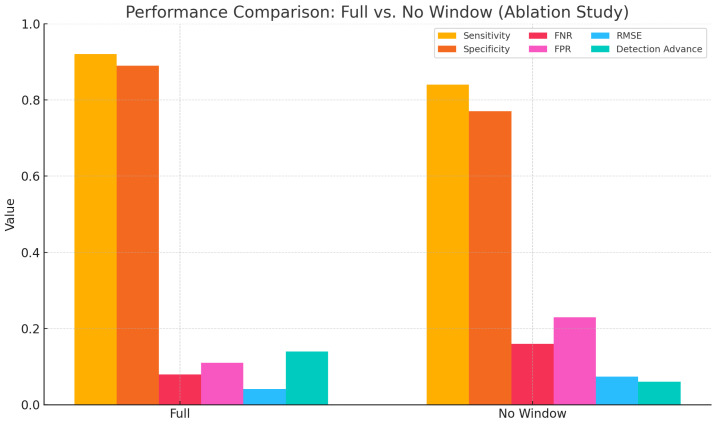
Comparison of six performance metrics between the full model and the model without the sliding window mechanism. The results highlight the significant role of the window structure in improving both accuracy and stability.

To further evaluate the impact of the sliding window mechanism on detection stability, an ablation experiment was conducted. In this configuration, the sliding window used for computing both marginal spectrum entropy (MSE) and the trend slope was removed. Instead, single-point values were extracted globally for decision-making. Compared to the complete model, this simplified version lacked local smoothing and statistical support, which may result in detection fluctuations and increased sensitivity to noise. As shown in Table 7, the removal of the sliding window led to a noticeable degradation in performance. Specifically, the root mean square error (RMSE) increased to 0.074 s, the detection advance decreased to 0.06 s, and both the false negative rate (FNR) and false positive rate (FPR) worsened. These results indicate that the sliding window plays a critical role in suppressing local signal fluctuations and enhancing the stability of feature representation. To further illustrate the contribution of the sliding window across various performance metrics, a comparative bar chart is presented in Figure 9. The figure clearly demonstrates that the removal of the sliding window led to overall declines in detection robustness and accuracy.

These findings further demonstrate that the sliding window not only enhances the smoothness of feature extraction but also maintains temporal resolution while improving model robustness under low-amplitude, fluctuating signals. Its structural support is particularly critical when dealing with slow activations and non-abrupt myoelectric patterns.

#### 4.2.2. Discussion

A novel muscle activation onset detection method based on the fusion of marginal spectrum entropy (MSE) and trend slope analysis was proposed, and its superior sensitivity and temporal accuracy under slow activation conditions were demonstrated through comparative experiments with mainstream methods. Existing techniques, such as fixed thresholding, Teager–Kaiser energy operator (TKE), root mean square (RMS) averaging, and wavelet-based energy decomposition, generally rely on fixed amplitude thresholds, instantaneous energy variations, or frequency domain energy distributions. These methods are typically effective in detecting abrupt or rapid activations. However, under conditions involving gradually increasing activations, slowly rising signals, or low signal-to-noise ratios, such approaches often suffer from delayed triggering or missed detections. In contrast, the proposed method leverages the sensitivity of MSE to frequency complexity and the precise tracking ability of trend slope analysis for slow-varying patterns. This fundamental distinction in methodological design enhances robustness and adaptability in non-abrupt activation scenarios, making the approach particularly suitable for progressive control tasks such as gait initiation, rehabilitation training, and functional electrical stimulation. Despite the promising results, several limitations regarding the generalizability of the method should be acknowledged. The evaluation was conducted on only 20 sEMG signal samples, all collected from the soleus muscle during natural walking conditions. The soleus, as a primary plantar flexor, exhibits relatively slow activation dynamics, rendering it a suitable test case for the proposed approach. However, significant differences exist across muscle groups in terms of morphology, fiber composition, and activation rhythms. Therefore, whether the proposed method maintains its performance on other muscles—such as the rectus femoris, biceps femoris, or forearm muscle groups—remains to be further investigated. Moreover, the limited sample size restricts the assessment of the method’s generalization under broader variations, including inter-subject diversity, changes in posture, and external load conditions. Future work may involve expanding the dataset to include multiple muscles and tasks, and incorporating transfer learning strategies across subjects to enhance the model’s adaptability and practical scope. Although the proposed method achieves higher precision and lower FNR, its offline nature limits its use in closed-loop real-time applications. Future work will explore an incremental entropy update mechanism to enable low-latency deployment.

From a quantitative perspective, the proposed method enables muscle onset detection 0.14∼0.16 s earlier, on average, than the baseline methods. Although this lead time appears modest in engineering terms, it holds critical importance in real-time control systems. For instance, in neurorehabilitation robotics, even a 0.1 s increase in system response delay may cause gait asynchrony or incorrect muscle stimulation, thereby negatively affecting patient recovery. In movement feedback control, earlier intention recognition significantly enhances system responsiveness and naturalness. Such advantages are particularly relevant in time-sensitive clinical applications such as early-stage post-stroke rehabilitation, assistance for muscular weakness, and brain-computer interface-based interventions. Additionally, a preliminary interpretation of “slow activation” in sEMG signals was explored. In practical acquisition scenarios, slow activation does not merely refer to gradual amplitude changes but rather describes an activation process characterized by smooth transitions in both time and frequency domains, lacking clear-cut boundaries or abrupt features. These types of signals are more vulnerable to background noise, asynchronous activations, and inter-individual variability, which pose challenges for traditional detection algorithms. By combining spectral entropy with slope analysis, the proposed method captures weak yet continuous trends embedded in the signal, thereby enabling the identification of subtle precursors to activation onset. This mechanism not only proved effective in the soleus muscle scenario presented here but also holds the potential for extension to more complex conditions involving neuromuscular impairments or fatigue.

#### 4.2.3. Parameter Sensitivity and Justification

To validate the rationality of the parameter settings used in this study, a sensitivity analysis was conducted focusing on two key parameters, that is, the sliding window length and the resolution of marginal spectrum entropy (MSE) computation. In the main experiments, the window length was set to 128 ms with a 50% overlap. This choice was guided by previous literature recommending similar values for extracting mid-to-low-frequency features in EMG signals, as it balances temporal resolution with sufficient spectral information. To assess robustness, we varied the window length between 96 ms and 192 ms and observed that the model yielded the most stable performance around 128 ms, with the lowest RMSE and false negative rate (FNR), confirming its effectiveness in capturing gradual signal transitions. For MSE calculation, we employed a Hilbert–Huang transform (HHT)-based marginal entropy measure. The process involved decomposing the signal using empirical mode decomposition (EMD), computing the instantaneous spectrum, and estimating the marginal spectral density, followed by normalized entropy calculation. The number of modes was limited to a maximum of six, and frequency components were discretized using equal-width binning for entropy estimation. Experimental results demonstrated that this configuration offered strong trend-tracking capabilities for slowly activated signals and provided better stability and interpretability than traditional multiscale entropy or short-time Fourier-based approaches. These parameter choices are clarified in the Methods section and further analyzed in the Results section.

#### 4.2.4. Limitation and Future Work

Although the proposed muscle activation onset detection method based on marginal spectrum entropy (MSE) has demonstrated promising performance in slow activation scenarios, several limitations may affect its generalizability and broader applicability. First, the dataset used in this study is relatively limited in both sample size and diversity. The current experiments include only 20 sEMG sequences collected from 10 male and 10 female healthy adults, with recordings performed during natural walking and limited to the right soleus muscle. While the soleus, a muscle rich in slow-twitch fibers, is well-suited for evaluating the detection performance under slow activation conditions, focusing exclusively on this muscle restricts the extrapolation of results to other muscle groups or activation patterns. Second, the experimental task—level-ground walking—represents a relatively simple locomotor activity and does not cover more complex or varied movement types, such as upper limb motions, high-speed activities (e.g., running), or postural transitions. As such, the applicability of the proposed method in scenarios involving rapid muscle recruitment or co-contraction remains to be further validated. Third, all participants were healthy, right-foot dominant, and free from any history of neuromuscular disorders, which may introduce selection bias and limit the clinical relevance of the findings to populations such as stroke survivors, elderly individuals with sarcopenia, or patients with neurological impairments. To address these limitations, future work will focus on three main directions. First, the dataset will be expanded to include additional muscle groups (e.g., rectus femoris, biceps brachii, tibialis anterior) and a wider range of motor tasks (e.g., sit-to-stand transitions, reaching, stair climbing), in order to comprehensively evaluate the anatomical and functional generalizability of the method. Second, the subject population will be broadened to include elderly individuals, patients with neuromotor impairments, and clinical populations at different stages of rehabilitation, to assess robustness and translational potential. Third, domain adaptation and transfer learning techniques will be explored to systematically enhance cross-subject generalization, reduce reliance on subject-specific calibration, and improve performance in real-world deployment settings.

## 5. Conclusions

Accurate detection of muscle activation onset in surface electromyography (sEMG) analysis serves as a critical prerequisite for applications in human–machine interaction, rehabilitation control, and motion recognition. This requirement becomes particularly challenging under neuromuscular impairment or fatigue conditions, where muscle activations often exhibit gradual signal transitions without clear abrupt changes, significantly reducing the effectiveness of traditional threshold-based or energy-based detection algorithms. To address this challenge, a novel detection approach was proposed, which integrates marginal spectrum entropy (MSE) with trend slope analysis, aiming to enhance detection sensitivity and temporal precision for slowly activated signals while overcoming the limitations associated with abrupt change dependency in existing methods. The proposed method evaluates the evolving trend of MSE within a sliding window and combines it with local slope analysis to determine the activation point, thereby eliminating the reliance on fixed amplitude thresholds. This framework is particularly suitable for scenarios characterized by slow signal amplitude increases and subtle frequency-domain transitions. Benchmark comparisons were conducted against four established methods, including fixed thresholding, the Teager–Kaiser energy (TKE) operator, RMS-based windowing, and wavelet-based energy analysis. In addition, multiple ablation experiments were designed to assess the independent contributions of the MSE module, trend slope module, and sliding window mechanism. Experimental results demonstrated that the proposed method achieved an average detection advance of 0.14∼0.16 s on a soleus sEMG dataset, outperforming all four baseline methods. For instance, the proposed method improved the detection advance by approximately 0.14 s compared to the fixed threshold method and by 0.12 s over the TKE operator method. In terms of performance metrics, the method yielded the highest sensitivity (0.92) and specificity (0.89) across all 20 signal samples, while also achieving the lowest false negative rate (FNR = 0.08), false positive rate (FPR = 0.11), and root mean square error (RMSE = 0.041 s). By contrast, the TKE and RMS methods exhibited elevated FNR and FPR values in the range of 0.17∼0.20, with RMSE generally exceeding 0.06 s, indicating insufficient accuracy in temporal localization. Additionally, a structured interpretation of “slow activation” in sEMG signals was addressed. Such activations are characterized not solely by gradual amplitude changes in the time domain but also by the absence of sharp frequency-domain transitions, ambiguous boundaries, and increased susceptibility to background noise. These properties often cause conventional algorithms to misclassify or delay the onset detection. The proposed MSE and slope fusion strategy, through modeling both spectral complexity and temporal trends, effectively captures subtle but continuous activation precursors, enabling early and reliable detection of ambiguous onset points.

## Figures and Tables

**Table 1:** The onset times for 20 sEMG sets detected by the methods.

Signal ID	Method A (s)	Method B (s)	Method C (s)
S01	1.10	1.40	1.26
S02	0.64	0.93	0.78
S03	0.65	0.65	0.70
S04	0.60	0.83	0.86
S05	0.42	0.59	0.48
S06	0.78	0.79	0.96
S07	0.90	1.00	1.18
S08	0.60	0.72	0.72
S09	0.54	0.69	0.68
S10	0.55	0.56	0.56
S11	0.53	0.59	0.72
S12	0.50	0.63	1.18
S13	0.55	0.67	0.66
S14	0.54	0.74	0.72
S15	0.49	0.64	0.62
S16	0.54	0.67	1.04
S17	0.50	0.70	0.54
S18	0.73	0.79	0.78
S19	0.53	0.68	0.56
S20	0.42	0.70	0.68

**Table 2:** Male participants’ anthropometrics, exercise habits, and caffeine intake on the day of the trial.

Participant	Gender	Age	Height (cm)	Weight (kg)	Muscle Injury	Had Coffee Today?
M01	M	25	178	75	No	No
M02	M	25	176	70	No	No
M03	M	24	178	70	No	No
M04	M	23	180	75	No	No
M05	M	25	175	75	No	No
M06	M	25	175	68	No	No
M07	M	25	170	50	No	No
M08	M	26	175	65	No	No
M09	M	27	175	73	No	No
M10	M	25	175	72	No	No

**Table 3:** Female participants’ anthropometrics, exercise habits, and caffeine intake on the day of the trial.

Participant	Gender	Age	Height (cm)	Weight (kg)	Muscle Injury	Had Coffee Today?
F01	F	24	172	60	No	No
F02	F	24	160	55	No	No
F03	F	25	160	55	No	No
F04	F	24	168	65	No	No
F05	F	27	167	53	No	No
F06	F	22	164	50	No	No
F07	F	25	165	57	No	No
F08	F	22	165	50	No	No
F09	F	27	163	55	No	No
F10	F	26	163	52	No	No

**Table 4:** Basic information on subjects (Mean ± SD).

Gender	Age (years)	Height (cm)	Weight (kg)
Male	25.0±1.1	175.7±2.7	69.3±7.5
Female	24.6±1.8	164.7±3.7	55.2±4.6

**Table 5:** Performance comparison of different methods for muscle onset detection.

Method	Sensitivity	Specificity	FNR	FPR	RMSE (s)	Detection Advance (s)
Threshold	0.81	0.78	0.19	0.22	0.084	0.00
TKE	0.83	0.80	0.17	0.20	0.069	0.02
RMS	0.82	0.77	0.18	0.23	0.073	0.01
Wavelet	0.84	0.81	0.16	0.19	0.066	0.03
Proposed	**0.92**	**0.89**	**0.08**	**0.11**	**0.041**	**0.14**

**Table 6:** Performance comparison of ablation variants in muscle onset detection.

Method	Sensitivity	Specificity	FNR	FPR	RMSE (s)	Detection Advance (s)
MSE + slope	**0.92**	**0.89**	**0.08**	**0.11**	**0.041**	**0.14**
energy + slope only	0.85	0.80	0.15	0.20	0.066	0.07
MSE only	0.86	0.82	0.14	0.18	0.060	0.09

**Table 7:** Performance comparison after removing the sliding window mechanism.

Method	Sensitivity	Specificity	FNR	FPR	RMSE (s)	Detection Advance (s)
with window	**0.92**	**0.89**	**0.08**	**0.11**	**0.041**	**0.14**
global value	0.84	0.77	0.16	0.23	0.074	0.06

## Data Availability

The data presented in this study are available upon request from the corresponding author.
